# Molecular Detection of *Bartonella* Species in Rodents Residing in Urban and Suburban Areas of Central Thailand

**DOI:** 10.3390/microorganisms9122588

**Published:** 2021-12-15

**Authors:** Phirabhat Saengsawang, Serge Morand, Marc Desquesnes, Sarawut Yangtara, Tawin Inpankaew

**Affiliations:** 1 Akkhraratchakumari Veterinary College, Walailak University, Nakhon Si Thammarat 80161, Thailand; phirabhat.s@gmail.com or; 2 CNRS ISEM—CIRAD-ASTRE, Montpellier University, 34090 Montpellier, France; serge.morand@umontpellier.fr; 3 Faculty of Veterinary Technology, Kasetsart University, Bangkok 10900, Thailand; 4 InterTryp, Université de Montpellier CIRAD-IRD, 34090 Montpellier, France; marc.desquesnes@cirad.fr; 5 Ecole Nationale Vétérinaire de Toulouse (ENVT), 31300 Toulouse, France; 6 Department of Companion Animal Clinical Science, Faculty of Veterinary Medicine, Kasetsart University, Bangkok 10900, Thailand; great_vt14@hotmail.com; 7 Department of Parasitology, Faculty of Veterinary Medicine, Kasetsart University, Bangkok 10900, Thailand

**Keywords:** *Bartonella*, *Rattus*, zoonosis, diversity, *gltA*, Thailand

## Abstract

*Bartonella* spp. are Gram-negative zoonotic bacteria transmitted to humans via various blood-sucking arthropods. Rodents have been identified as reservoir hosts of several zoonotic pathogens, including *Bartonella* spp. In Thailand, studies of *Bartonella* spp. in rodents from urban areas are limited; thus, a study in this area is necessary. The objectives of this study were to detect *Bartonella* spp. in rodents in Thailand and to compare the species’ distribution across different areas. In total, 70 blood samples from rodents in urban and suburban areas were tested for *Bartonella* spp. using a conventional polymerase chain reaction that targeted the citrate synthase (*gltA*) gene. All *Bartonella*-positive sequences were analyzed using polymorphism in order to build a phylogenetic tree. Approximately 38% of the rodents studied contained *Bartonella* DNA. Both *Rattus exulans* (Pacific rat) and *R. tanezumi* (Asian house rat) contained *Bartonella* spp. Four species of *Bartonella* were detected in blood samples: *B. tribocorum*, *B. phoceensis*, *B. grahamii*, and *B. rattimassiliensis*. In addition, eight Pacific rats contained the *B. kosoyi*–*B. tribocorum* complex. *Bartonella phoceensis* and *B. tribocorum*–*B. kosoyi* complexes were found in a specific habitat (*p* < 0.05). Interestingly, only seven haplotypes were identified in the sequences analyzed, and only haplotype A was found in both rodent species. Finally, a monitoring program for zoonotic *Bartonella* infection, especially the *B. kosoyi*–*B. tribocorum* complex, *B. phoceensis*, *B. grahamii*, and *B. rattimassiliensis* should be established, especially in high-risk areas.

## 1. Introduction

*Bartonella* spp. are Gram-negative intraerythrocytic bacteria [1] that have been rearranged into the alpha-proteobacteria [2] and are transmitted by blood-sucking arthropods [3]. Several species of *Bartonella* have been confirmed as zoonotic pathogens, including *B. henselae*, *B. clarridgeiae*, *B. elizabethae*, *B. grahamii*, *B. koehlerae*, *B. quintana*, *B. washoensis*, and *B. vinsonii* [4]. New members of the genus *Bartonella* are being found continuously [5], and more than 35 species have had their whole-genome sequences identified. To date, around 6 of the 20 rodent-adapted *Bartonella* spp. are zoonotic pathogens [6] that are important in medical and veterinary medicine [7]. Several types of animals are confirmed as hosts transmitting *Bartonella* spp., including cats [4,8], dogs [1,4], cattle [9,10], and rodents [11,12]. Furthermore, *Bartonella* spp. have been detected in rodents in several continents, including Asia [13], Africa [14], Europe [7], Americas [12,15], and Australia [16].

Some rodents have been suspected of being a source of zoonotic pathogens transmitted to humans [17], such as *Rickettsia* spp., *Leptospira* spp., *Coxiella burnetii*, *Orientia tsutsugamushi*, and *Bartonella* spp. [18]. From a public health viewpoint, there could be an increase in human cases due to infection by rodent-borne *Bartonella* spp. from outdoor activities [6] and other indirect contact. Changing land use and the sharing of habitat among rodents, animals, and humans have been identified as causes of zoonotic infection [19,20]. Several *Bartonella* spp. that have been associated with specific species of rodent could be the causative agents of *Bartonella*-related diseases in humans, such as endocarditis, lymphadenopathy, and some neurological abnormalities [11,21].

*Bartonella* infection in rodents frequently shows persistent and subclinical bacteremia [6,18]. Approximately 90 species of rodent have *Bartonella* spp. [18] variants in abundance, and at least 22 species of *Bartonella* have been found in rodents [6]. *Bartonella doshiae* [22], *B. elizabethae* [23], *B. grahamii* [24], *B. rochalimae* [25], *B. tamiae* [26], *B. tribocorum* [27], *B. vinsonii* subsp. *arupensis* [28], and *B. washoensis* [29] have been reported as the main *Bartonella* spp. found in rodents that cause human infections. Compared with other mammals, the different level of infection and high genetic diversity of *Bartonella* spp. in rodents has been noted [5].

In 2010, Thai febrile patients showed evidence of zoonotic species of *Bartonella* based on a molecular detection [26,30]. In Thailand, the study of zoonotic *Bartonella* spp. has been mainly conducted in companion animals [31,32,33] and their ectoparasites [34]; however, studies involving rodents are limited, and additional studies in urban areas are needed [35]. With regard to the Thai government’s “One Health” approach to humans, animals, and vectors, further studies on *Bartonella* are also necessary [34]. The current study therefore aims to survey the prevalence of *Bartonella* infection in rodents and to compare the species of *Bartonella* in areas of different characteristics (urban and suburban environments).

## 2. Materials and Methods

### 2.1. Sample Size

The sample size was calculated using an equation for the infinite population proportion [36] and a prevalence (*p*), taken from a previous study in Thailand [19]. Bangkok and Nakhon Sawan provinces were defined as urban and suburban areas, respectively. Hence, the previous proportion (*p* = 4.38%) was re-calculated from data on settlement and rain-fed areas derived from the previous study. For the sample size calculation, the maximum tolerated error (*d*) and alpha (*α*) were set at 5%. In total, the calculated sample size was 65 rodents based on the following equation:

$$n=\frac{Z_{1-\frac{\alpha }{2}\;}^{2}\times p\times \left(1-p\right)}{d^{2}}$$

### 2.2. Sample Collection

**Figure 1:**
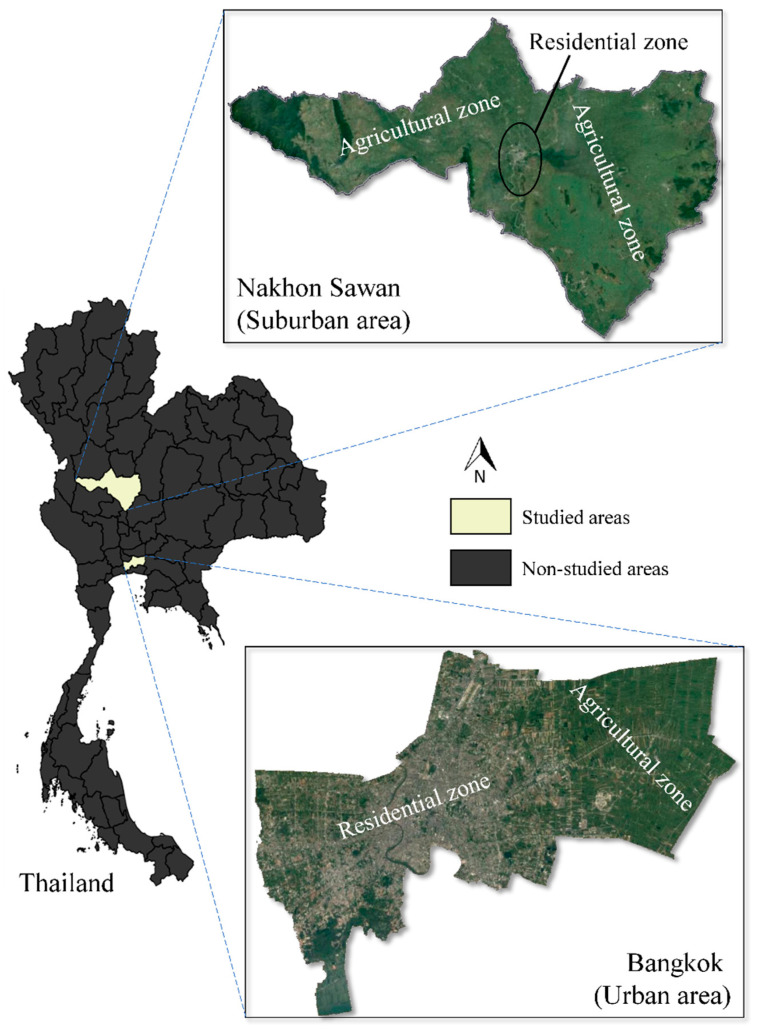
Sample collection sites and their geographic characteristics.

The sampling was approved by the Kasetsart University Institutional Animal Care and Use Committee, Bangkok, Thailand, under the Ethical Review Board of the Office of the National Research Council of Thailand (NRCT; approval ID: ACKU63-VET-048). This study was a cross-sectional survey of rodents trapped from fields in two provinces (Bangkok and Nakhon Sawan), as shown in Figure 1. The rodents were trapped between 2011 and 2013. All trapped rodents were classified based on external morphological characteristics. Three milliliters of blood were collected from each sample using aseptic cardiac puncture and were kept in a sterile EDTA tube. Euthanasia was conducted using chloroform inhalation after blood collection. All blood samples were stored at −20 °C until DNA extraction. Additionally, the standard procedures applied by the laboratory in this study followed the verification of the Institutional Biosafety Committee (IBC), Faculty of Veterinary Medicine, Kasetsart University.

### 2.3. DNA Extraction from Whole Blood

Two hundred microliters of whole blood was extracted for genomic DNA testing using a commercial extraction kit (FavoPrep^TM^ Blood DNA Extraction Mini Kit, Favorgen Biotech Corporation, Pingtung, Taiwan). The extraction protocol was performed according to the manufacturer’s instructions, and 100 µL of nuclease-free water was used as the elution solution. The extracted DNA was kept at −20 °C until a polymerase chain reaction (PCR) was performed.

### 2.4. Bartonella Detection Using Polymerase Chain Reaction

*Bartonella* spp. were detected using conventional PCR. BhCS.781p (5′-GGGGACCAGCTCATGGTGG-3′) and BhCS.1137n (5′-AATGCAAAAAGAACAGTAAACA-3′) were used to target the citrate synthase (*gltA*) gene of *Bartonella* spp. [37]. The targeted 379 bp fragment was suspected to be a *Bartonella*-positive blood sample, and the conditions of amplification were controlled using a thermocycler (Mastercycler^®^ Nexus Gradient, Eppendorf, Hamburg, Germany). In total, 25 µL of PCR mixture (0.2 mM of each dNTPs, 1X of *Taq* reaction buffer with MgSO_4_, 4 pmol/µL of each primer, 0.04 U/µL of *Taq* DNA polymerase, 0.8% of dimethyl sulfoxide, and 3 µL of DNA template) were run for PCR detection and commercial *Taq* DNA polymerase was used (*Taq* DNA Polymerase, Applied Biological Materials (ABM^®^ Inc., Richmond, BC, Canada). The running of PCR for BhCS.781p and BhCS.1137n was performed as follows: 5 min at 95 °C for initial denaturation, 35 repeated cycles of denaturation (95 °C, 20 s), annealing (51 °C, 30 s), and elongation (72 °C, 2 min). The last elongation was conducted at 72 °C for 5 min. *Bartonella henselae* strain Houston-1 DNA and nuclease-free water were used as the positive and negative controls, respectively. The amplified products were kept at 4 °C until gel electrophoresis.

### 2.5. Gel Electrophoresis and Purification

Twenty microliters of the amplified product was run in 1.5% agarose gel under a 0.5X tris-acetate EDTA (TAE) buffer for 45 min at 100 V, and UltraPower^TM^ Nucleic Acid Stain (BioTeke Corporation, Wuxi, China) was used for DNA staining. DNA visualization was performed using an ultraviolet illuminator (Gel Doc InGenius, SYNGENE, Frederick, MD, USA) and a 100-bp DNA ladder was used as the DNA size marker (Enzynomics, Daejeon, South Korea). The 379 bp band was cut and purified using a DNA purification kit (Gel and PCR Purification System, BioFACTTM, Daejeon, South Korea). The running protocol followed the manufacturer’s instructions. Forty microliters of eluted purified DNA fragments were sent to a commercial sequencing unit using Sanger’s sequencing technology (Macrogen^®^, Seoul, Korea).

### 2.6. Analysis of DNA Sequence

The obtained DNA sequences were trimmed using the Chromatogram Explorer Lite version 5.0.2 software (http://www.dnabaser.com, accessed on 20 November 2021) under the default low-quality end trimming conditions (75% of good bases, 18 bases of window length, and 25 quality value (QV) of good base). The trimmed DNA chromatograms were edited using the SnapGene^®^ Viewer version 5.3.2 software (https://www.snapgene.com/snapgene-viewer, accessed on 20 November 2021) and analyzed using BLASTn (https://blast.ncbi.nlm.nih.gov/Blast.cgi, accessed on 20 November 2021). A phylogenetic tree was constructed using the neighbor-joining method based on a proper substitution model with 1000 bootstrapping replications in the Molecular Evolutionary Genetics Analysis (MEGA) version X software (https://www.megasoftware.net, accessed on 20 November 2021). *Bartonella* positive sequences were analyzed for polymorphism based on the number of variable sites (VS), the proportion of G + C content (GC), the number of haplotypes (h), the average number of nucleotide differences (*k*), haplotype diversity (Hd), and nucleotide diversity (*π*) using the DNA Sequence Polymorphism (DnaSP) version 6.12.03 software (http://www.ub.edu/dnasp, accessed on 20 November 2021). Then, the sequences were analyzed based on a median-joining network using the Population Analysis with Reticulate Trees (PopART) version 1.7 software (http://popart.otago.ac.nz/index.shtml, accessed on 20 November 2021) with the default setting (epsilon = 0). All *Bartonella*-matched sequences were submitted to GenBank with accession numbers: OK381826–OK381850.

### 2.7. Statistical Analysis

The data were presented using descriptive statistics (mean and standard deviation). Prevalence was calculated, and the Wilson score interval method was used to estimate the 95% confidence interval of prevalence [38]. Associated factors were analyzed using Chi-square or Fisher’s exact test. All statistical analyses were performed using the 95% confidence interval (CI), and *p* < 0.05 was considered the significant level. All statistical analyses were performed using the R programming language version 4.0.2 [39].

## 3. Results

### 3.1. Bartonella Species in Rodents

In total, 70 rodents were trapped in the two different environments: 30 *Rattus exulans* in urban (Bangkok) and 40 *R. tanezumi* in suburban (Nakhon Sawan). Of these, 27 (38.57%; 95% CI = 28.05–50.28%) had *Bartonella* DNA in their blood samples. Overall, no associated factors of *Bartonella* infection were identified in the rodents (Table 1). 

Of the Pacific rat blood samples, 13 (43.33%; 95% CI = 27.38–60.80%) were positive for *Bartonella gltA* fragments. Additionally, 14 Asian house rats (35.00%: 95% CI = 22.13–50.49%) had a *gltA* fragment of *Bartonella* spp. The BLAST results revealed a *B. kosoyi*–*B. tribocorum* complex (11.43%; 95% CI = 5.91–20.96%) and *B. phoceensis* (20.00%%; 95% CI = 12.30–30.82%). All BLASTn results are presented in Table 2.

Eight sequences from *R. exulans* closely matched the *B. kosoyi* sequences (% identity = 99.36–100%) isolated from black rats (*R. rattus*) (CP031843) and *B. tribocorum* sequences (% identity = 99.36–100%) isolated from humans (HG969192). Surprisingly, the results of BLASTn for both *B. kosoyi* and *B. tribocorum* had similarity percentages; however, there were differences in non-compatible positions. Hence, these sequences (*n* = 8) were called a *B. kosoyi*–*B. tribocorum* complex. Interestingly, 14 sequences from *R. tanezumi* and *R. exulans* were similar to the *B. phoceensis* sequence (% identity = 97.94–100%) isolated from brown rats (*R. norvegicus*) (AY515126). In more minor findings, two other sequences (one from an Asian house rat and the other from a Pacific rat) matched *B. rattimassiliensis* (%identity = 100%; JX158359) and *B. grahamii* (%identity = 100%; GU056195), respectively. Comparing the two species (*B. phoceensis* and the *B. kosoyi*–*B. tribocorum* complex) and areas (Bangkok and Nakhon Sawan), there were significant differences in the proportion of *Bartonella* spp. Overall, *B. phoceensis* was found mostly in Nakhon Sawan, while the *B. kosoyi*–*B. tribocorum* complex was found only in Bangkok.

### 3.2. Phylogenetic Tree and Polymorphism Based on gltA Sequences

**Figure 2:**
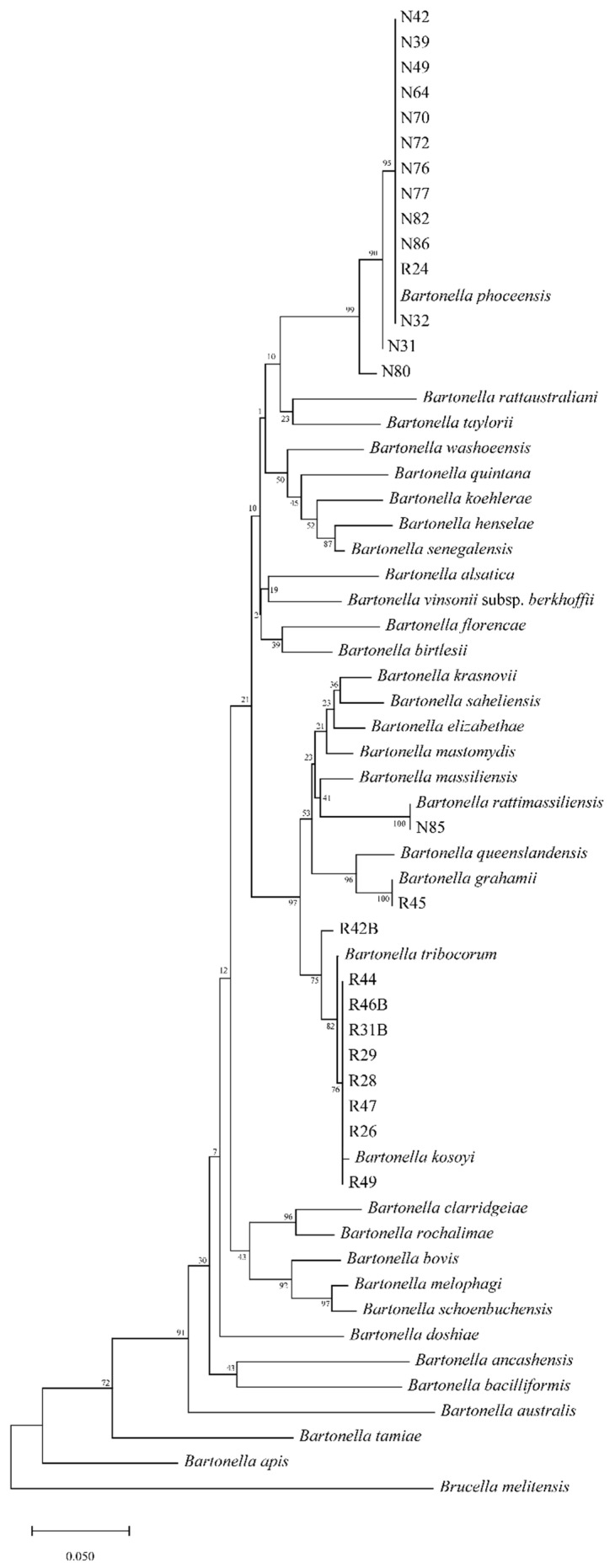
Phylogenetic tree of *Bartonella gltA* sequences obtained from rodents in this study.

**Figure 3:**
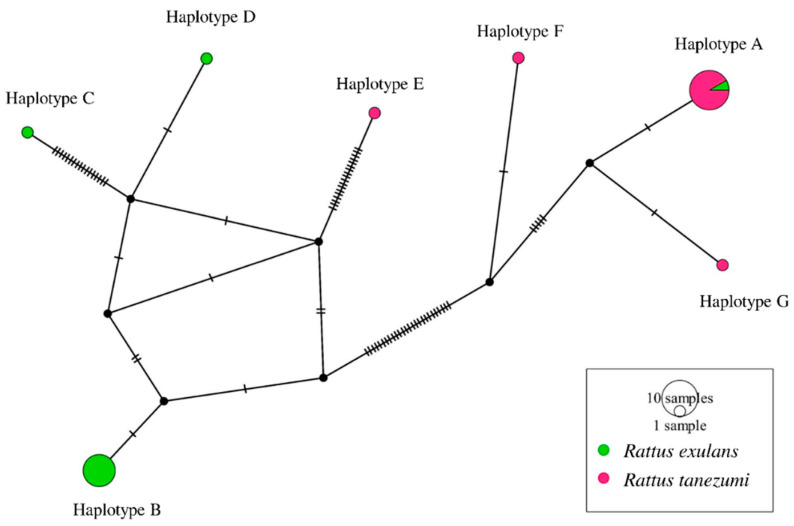
Median-joining network of *gltA* haplotypes from two different rodent species in Thailand.

Sequencing of the *gltA* fragments identified five species of *Bartonella*; the phylogenetic tree of the *Bartonella* positive sequences is presented in Figure 2. The tree had two main species complexes, consisting of a *B. phoceensis* complex (*n* = 14) and a *B. kosoyi*–*B. tribocorum* complex (*n* = 9). However, two sequences in the *B. phoceensis* complex were separate from the others. In the same way, in the *B. tribocorum*–*B. kosoyi* complex, one sequence was separate from the others. The polymorphism information of the partial *gltA* sequences that matched the *B. kosoyi*–*B. tribocorum* complex and *B. phoceensis* is presented in Table 3. Additionally, the variable position (singleton and parsimony-informative sites) is presented in Table 4. For these two complexes, the median-joining network is illustrated in Figure 3. Seven different haplotypes among 25 *gltA* sequences (*n* = 13 for *R. exulans* and *n* = 14 for *R. tanezumi*) showed *π* = 0.06471 ± 0.00617, Hd = 0.687 ± 0.071, and *k* = 19.61. Only haplotype A (12 sequences) showed different species of rodent that had *B. phoceensis*. However, other haplotypes (B-G) revealed specific *Bartonella* spp. Comparing the with and without reference sequences, those that matched *B. kosoyi* and *B. tribocorum* were clearly different from the references (CP031843 and HG969192). In contrast, the diversity difference between the with and without reference groups in *B. phoceensis* was not different from the previous finding.

## 4. Discussion

In this study, the trapped rodents belonged to two species: *R. exulans* and *R. tanezumi*. In Thailand, various species of rodent were identified, including *Rattus* spp., *Bandicota* spp., *Leopoldamys* spp., *Mus* spp., and *Niviventer* spp. [40]. *Rattus* spp. was found in suburban areas in the current study. Of these, *R. tanezumi* and *R. exulans* were reported as major species of *Rattus* in Thailand [40]. Interestingly, *R. tanezumi* is a synanthropic rodent species mostly found in suburban environments, including residential and agricultural areas [41]. In urban areas, some species of *Rattus* were also reported such as *R. norvegicus* and *R. rattus* [35]. Due to the increase in human–rodent contact, attention has been focused on various emerging diseases caused by novel pathogens [42]. Several rodent-borne pathogens can cause various human diseases, such as hantavirus, *Borrelia* spp., *Toxoplasma gondii*, *Yersinia pestis*, *Bartonella* spp., *Leptospira* spp., and *Coxiella burnetii* [43].

Many rodents in the current study had *Bartonella* spp. in their blood samples. Importantly, rodents have been mentioned as a major source of *Bartonella* infection in humans [44]. Rodent-borne *Bartonella* spp. have been discovered globally, and the rodent-adapted *Bartonella* spp. have high diversity [6]. Partial sequences of the *gltA* gene revealed five species of *Bartonella*: *B. tribocorum*, *B kosoyi*, *B. phoceensis*, *B. grahamii*, and *B. rattimassiliensis*. Of these, three species have been reported as human pathogens [30,45]. The overall prevalence of *Bartonella* spp. in the current study differed from other studies in Malaysia [46] and China [13]; however, the prevalence of a study in Chile [12] was similar to the prevalence of the current study. Most studies in Thailand have reported *Bartonella* spp. being frequently isolated from *R. rattus* [20,47,48]. The prevalence of *Bartonella* spp. in *R. tanezumi* in the current study was similar to that found in a study in Singapore [49]; however, it contrasts with many other studies [17,50,51]. *Bartonella rattimassiliensis* and *B. phoceensis* were positive in *R. tanezumi* blood samples, which is a result similar to those of studies in Malaysia [46], Indonesia [52], Vietnam [50], and Singapore [49]. In addition, two zoonotic *Bartonella* spp. (*B. tribocorum* and *B. grahamii*) were detected in *R. exulans* blood samples, which was similar to other studies in Thailand [17,48,53]. Nevertheless, in the current study, the prevalence of *Bartonella* spp. in *R. exulans* differed from other studies [17,47,48,49,50,53,54,55]. Particularly in urban habitats, *Bartonella* infection risk in humans increases from contact among humans, rodents, and ectoparasites [56]. In Thailand, rodent lice and fleas have been reported to carry *Bartonella* spp. and to circulate these pathogens in the rat population [47].

The previously reported prevalence of *Bartonella* spp. infection in rodents varied from 6% to 100% [18]. There are several factors related to the *Bartonella* prevalence rate in rodents, including habitat characteristics, body mass of rodents, age, rodent species, climate, rodent behavior, movement pattern, sampling method, and detection technique [11,18,35,46,57,58]. Additionally, varied levels of prevalence and *Bartonella* diversity were mentioned as common in rodents that had caused the heterogenous distribution of pathogens [59]. Even if there were no associated factor related to *Bartonella* infection in the studied rodents, the comparison between species of rodent and *Bartonella* revealed specificity. However, there was a higher infection rate in the urban area (Bangkok). Furthermore, the cool season had a higher rate of *Bartonella* infection than the warm season. As the preferred habitat of each rodent species differed, the detected *Bartonella* spp. might have been affected by characteristics of the habitat in the current study. Moreover, the size and structure of the rat population, referred to as “ecological factors”, related to *Bartonella* prevalence and diversity, particularly in the urban environment [56,59,60].

Inferring detected species, 11 of the 25 rodents carried zoonotic *Bartonella* spp. One *R. exulans* was carrying *B. grahamii*, which has been defined as a human pathogen and *Ctenophthalamus nobilis* has been suspected of being a possible vector [24]. Additionally, *B. tribocorum*, a zoonotic species [27], was also detected in one *R. exulans*. Interestingly, rodents have adapted to promote *Bartonella* spp. transmission to humans; in addition, *Polyplax spinulosa* and *Xenopsylla cheopis* have been mentioned as vectors of this species [27]. In other results, one *R. tanezumi* provided evidence of *B. rattimassiliensis*, which has also been identified as a zoonotic species carried by *P. spinulosa*, *Haemophysalis longicornis*, and *Hoplopleura pacifica* [61]. *Bartonella phoceensis* was found in both *R. tanezumi* and *R. exulans*. *Bartonella phoceensis* has not been reported as causing human infection, and *P. spinulosa* has been suggested as a vector carrying this non-zoonotic species [61]. Interestingly, eight of the nine *B. tribocorum*-positive *R. exulans* also matched a sequence of *B. kosoyi* with the same similarity and query coverage percentage but with differences in nucleotide substitution positions. The finding of *B. kosoyi* in *R. exulans* was similar to that in a study in Myanmar [55]. To date, there is no explanation of the relationship regarding *gltA* between *B. tribocorum* and *B. kosoyi*. However, the genome of *B. kosoyi* is closely related to *B. elizabethae* [62], which has been defined as a zoonotic species of *Bartonella* [23]. In addition, no evidence has been reported of *B. kosoyi* infection in humans, even though it has been isolated in rodents elsewhere [55,62,63].

The citrate synthase gene is widely used for *Bartonella* detection [64,65]. The citrate synthase gene was targeted for *Bartonella* detection in the current study, and seven haplotypes of partial *gltA* sequences were revealed. Several genetic events including mutation, demography, and recombination were factors regulating haplotype diversity [11]. Trimmed *gltA* sequences (approximately the 327 base pair) have been acclaimed for taxonomic classification in the genus *Bartonella* [66] and for distinguishing among subspecies and species [2,37,65,66]. However, an additional RNA-polymerase beta subunit (*rpoB*) gene has been noted to increase identification efficacy, especially for the classification of new species [66]. Remarkably, *gltA* sequencing was suggested as a common method for *Bartonella* diversity study in wild animals [6], although homologous recombination was an important point of this gene [67,68]. Compared with sequences in the NCBI database, the *gltA* sequences of *Bartonella* spp. have been continuously updated, and this has facilitated more species to be distinguished [47,65]. Furthermore, the citrate synthase gene showed synonymous amino substitutions, and it has been emphasized that *gltA* was an important gene for critical functions [65]. Importantly, sequencing in the current study aimed to detect species of *Bartonella* using a reliable *gltA* marker. All sequences had values of similarity percentage > 96%, indicating a full match for the species, based on the recommendations from the La Scola study [66].

Unfortunately, the detection of *Bartonella* spp. in the ectoparasites of the captured rodents was not included in the current study. However, there should be further study of infectious ectoparasites and ectoparasites in habitat environments in order to elucidate the dynamics of *Bartonella* spp. circulation in rodent populations. A complete explanation of the *gltA* gene and haplotype requires the analysis of the whole sequence of the *gltA* gene of *Bartonella*. A higher-level method should be used to fill this gap in knowledge, and full gene cloning and sequencing techniques should be considered for future studies.

## 5. Conclusions

In this study, the overall prevalence was 38.57% in rodents inhabiting areas of central Thailand (43.33% in *R. exulans* and 35% in *R. tanezumi*). Importantly, three zoonotic species were detected in the rodents’ blood samples (*B. tribocorum*, *B. grahamii* in *R. exulans*, and *B. rattimassiliensis* in *R. tanezumi*). Furthermore, *B. phoceensis* was identified as the major *Bartonella* spp. In this rodent population. Remarkably, *Bartonella phoceensis* and the complex of *B. tribocorum*–*B. kosoyi* were significant in the suburban (Nakhon Sawan province) and urban (Bangkok province) areas, respectively. Comparing polymorphism in *Bartonella*-positive and matched reference sequences found that the complex of *B. tribocorum*–*B. kosoyi* had more differences in nucleotide sequences than *B. phoceensis*. Seven haplotypes of the sequences analyzed were identified; however, only haplotype A showed infection in both *R. exulans* and *R. tanezumi*. The authors suggest monitoring zoonotic species of *Bartonella* infection in humans, particularly in workers in contact with rodents. Furthermore, updating the knowledge on *Bartonella*-related diseases should be supported in risk areas.

## Figures and Tables

**Table 1:** Factors associated with *Bartonella* infection in rodents.

Factor		Total	Positive	*p*-Value
Area	Urban (Bangkok)	30	13	0.65 ^a^
	Suburban (Nakhon Sawan)	40	14	
Rodent ^c^	Pacific rat (*Rattus exulans*)	30	13	0.65 ^a^
	Asian house rat (*Rattus tanezumi*)	40	14	
Season	Warm	58	20	0.19 ^b^
	Cool	12	7	

^a^ Chi-square test; ^b^ Fisher’s exact test; ^c^ All *R. exulans* found in Bangkok and all *R. tanezumi* found in Nakhon Sawan.

**Table 2:** Detected species of *Bartonella* based on *gltA* sequences.

Rodent	*Bartonella* spp.	n	Prevalence	
*R. exulans* (*n* = 30)	*B. kosoyi–B. tribocorum*	8	26.67%	14.18–44.45%
	*B. phoceensis*	1	3.33%	0.59–16.67%
	*B. grahamii*	1	3.33%	0.59–16.67%
	*B. tribocorum*	1	3.33%	0.59–16.67%
	*Bartonella* spp.	2	6.67%	1.85–21.32%
*R. tanezumi* (*n* = 40)	*B. phoceensis*	13	32.50%	20.08–47.98%
	*B. rattimassiliensis*	1	2.50%	0.44–12.88%

**Table 3:** Details of polymorphism of *B. kosoyi*–*B. tribocorum* and *B. phoceensis gltA* fragments.

*Bartonella* spp.	N	VS	GC	h	k	Hd ± SD	π ± SD
*B. kosoyi* ^a^	8	0	0.337	1	0.00	0.00	0.00
*B. kosoyi* ^b^	9	1	0.337	2	0.22	0.222 ± 0.166	0.00073 ± 0.00055
*B. tribocorum* ^a^	9	5	0.336	2	1.11	0.222 ± 0.166	0.00367 ± 0.00274
*B. tribocorum* ^b^	10	5	0.336	3	1.16	0.378 ± 0.181	0.00381 ± 0.00241
*B. phoceensis* ^a^	14	8	0.349	3	1.26	0.275 ± 0.148	0.00378 ± 0.00247
*B. phoceensis* ^b^	15	8	0.349	3	1.18	0.257 ± 0.142	0.00354 ± 0.00234

^a^ compared among sequences of this study; ^b^ compared among sequences of this study and reference (match) sequence; n = number of analyzed sequences; VS = number of variable sites; GC = proportion of G + C content; h = number of haplotypes; *k* = average number of nucleotide difference; Hd = haplotype diversity; *π* = nucleotide diversity; SD = standard deviation.

**Table 4:** Details of the variable positions of each *Bartonella*-positive group.

Species	Sequence	Accession Number	Length (bp)	Variable Position							
*B. kosoyi*	This study	OK381843-50	337	34 ^a^							
	Reference	CP031843		G→T							
*B. tribocorum*	This study	OK381826, 43-50	337	115 ^b^	220 ^a^	221 ^a^	271 ^a^	286 ^a^			
	Reference	HG969192		C→T	G→T	C→T	C→T	G→A			
*B. phoceensis*	This study	OK381828-41	341	47 ^a^	95 ^a^	104 ^a^	197 ^b^	207 ^a^	269 ^a^	300 ^a^	314 ^a^
	Reference	AY515126		C→T	C→T	T→C	G→T	G→A	C→T	T→C	C→T

^a^ Singleton variable site; ^b^ Parsimony informative site.

## Data Availability

The data are contained within the article.

## References

[1] Chomel B.B., Boulouis H.-J., Maruyama S., Breitschwerdt E.B. (2006). *Bartonella* spp. in pets and effect on human health. Emerg. Infect. Dis..

[2] Birtles R.J., Raoult D. (1996). Comparison of partial citrate synthase gene (*gltA*) sequences for phylogenetic analysis of *Bartonella* species. Int. J. Syst. Bacteriol..

[3] Regier Y., Rourke F.O., Kempf V.A.J. (2016). *Bartonella* spp.—A chance to establish One Health concepts in veterinary and human medicine. Parasit. Vectors.

[4] Álvarez-Fernández A., Breitschwerdt E.B., Solano-Gallego L. (2018). *Bartonella* infections in cats and dogs including zoonotic aspects. Parasit. Vectors.

[5] De Salvo M.N., Hercolini C., Arístegui E., Bruno A., Brambati D.F., Cicuttin G.L. (2020). *Bartonella* spp. associated with rodents in an urban protected area, Buenos Aires (Argentina). Comp. Immunol. Microbiol. Infect. Dis..

[6] Buffet J.-P., Kosoy M., Vayssier-Taussat M. (2013). Natural history of *Bartonella*-infecting rodents in light of new knowledge on genomics, diversity and evolution. Future Microbiol..

[7] Špitalská E., Minichová L., Kocianová E., Škultéty Ľ., Mahríková L., Hamšíková Z., Slovák M., Kazimírová M. (2017). Diversity and prevalence of *Bartonella* species in small mammals from Slovakia, Central Europe. Parasitol. Res..

[8] Chomel B.B., Kasten R.W. (2010). Bartonellosis, an increasingly recognized zoonosis. J. Appl. Microbiol..

[9] Boularias G., Azzag N., Gandoin C., Bouillin C., Chomel B., Haddad N., Boulouis H.J. (2020). *Bartonella bovis* and *Bartonella chomelii* infection in dairy cattle and their ectoparasites in Algeria. Comp. Immunol. Microbiol. Infect. Dis..

[10] Cherry N.A., Maggi R.G., Cannedy A.L., Breitschwerdt E.B. (2009). PCR detection of *Bartonella bovis* and *Bartonella henselae* in the blood of beef cattle. Vet. Microbiol..

[11] Gonçalves L.R., de Favacho A.R.M., Roque A.L.R., Mendes N.S., Fidelis O.L., Benevenute J.L., Herrera H.M., D’Andrea P.S., de Lemos E.R.S., Machado R.Z. (2016). Association of *Bartonella* species with wild and synanthropic rodents in different Brazilian Biomes. Appl. Environ. Microbiol..

[12] Müller A., Gutiérrez R., Seguel M., Monti G., Otth C., Bittencourt P., Sepúlveda P., Alabí A., Nachum-Biala Y., Harrus S. (2020). Molecular survey of *Bartonella* spp. in rodents and fleas from Chile. Acta Trop..

[13] Qin X.-R., Liu J.-W., Yu H., Yu X.-J. (2019). *Bartonella* species detected in rodents from eastern China. Vector Borne Zoonotic Dis..

[14] Kamani J., Morick D., Mumcuoglu K.Y., Harrus S. (2013). Prevalence and diversity of *Bartonella* species in commensal rodents and ectoparasites from Nigeria, West Africa. PLoS Negl. Trop. Dis..

[15] Jardine C., Appleyard G., Kosoy M.Y., McColl D., Chirino-Trejo M., Wobeser G., Leighton F.A. (2005). Rodent-associated *Bartonella* in Saskatchewan, Canada. Vector Borne Zoonotic Dis..

[16] Dybing N.A., Jacobson C., Irwin P., Algar D., Adams P.J. (2016). *Bartonella* species identified in rodent and feline hosts from island and mainland western Australia. Vector Borne Zoonotic Dis..

[17] Pangjai D., Maruyama S., Boonmar S., Kabeya H., Sato S., Nimsuphan B., Petkanchanapong W., Wootta W., Wangroongsarb P., Boonyareth M. (2014). Prevalence of zoonotic *Bartonella* species among rodents and shrews in Thailand. Comp. Immunol. Microbiol. Infect. Dis..

[18] Gutiérrez R., Krasnov B., Morick D., Gottlieb Y., Khokhlova I.S., Harrus S. (2015). *Bartonella* infection in rodents and their flea ectoparasites: An overview. Vector Borne Zoonotic Dis..

[19] Jiyipong T., Morand S., Jittapalapong S., Rolain J.-M. (2015). *Bartonella* spp. infections in rodents of Cambodia, Lao PDR, and Thailand: Identifying risky habitats. Vector Borne Zoonotic Dis..

[20] Castle K.T., Kosoy M., Lerdthusnee K., Phelan L., Bai Y., Gage K.L., Leepitakrat W., Monkanna T., Khlaimanee N., Chandranoi K. (2004). Prevalence and diversity of *Bartonella* in rodents of northern Thailand: A comparison with *Bartonella* in rodents from southern China. Am. J. Trop. Med. Hyg..

[21] de Favacho A.R.M., Andrade M.N., de Oliveira R.C., Bonvicino C.R., D’Andrea P.S., de Lemos E.R.S. (2015). Zoonotic *Bartonella* species in wild rodents in the state of Mato Grosso do Sul, Brazil. Microbes Infect..

[22] Vayssier-Taussat M., Moutailler S., Féménia F., Raymond P., Croce O., La Scola B., Fournier P.-E., Raoult D. (2016). Identification of novel zoonotic activity of *Bartonella* spp., France. Emerg. Infect. Dis..

[23] Daly J.S., Worthington M.G., Brenner D.J., Moss C.W., Hollis D.G., Weyant R.S., Steigerwalt A.G., Weaver R.E., Daneshvar M.I., O’Connor S.P. (1993). *Rochalimaea elizabethae* sp. nov. isolated from a patient with endocarditis. J. Clin. Microbiol..

[24] Birtles R.J., Harrison T.G., Saunders N.A., Molyneux D.H. (1995). Proposals to unify the genera *Grahamella* and *Bartonella*, with descriptions of *Bartonella talpae* comb. nov., *Bartonella peromysci* comb. nov., and three new species, *Bartonella grahamii* sp. nov., *Bartonella taylorii* sp. nov., and *Bartonella doshiae* sp. nov. Int. J. Syst. Bacteriol..

[25] Lin J.-W., Chen C.-Y., Chen W.-C., Chomel B.B., Chang C.-C. (2008). Isolation of *Bartonella* species from rodents in Taiwan including a strain closely related to “*Bartonella rochalimae*” from *Rattus norvegicus*. J. Med. Microbiol..

[26] Kosoy M., Morway C., Sheff K.W., Bai Y., Colborn J., Chalcraft L., Dowell S.F., Peruski L.F., Maloney S.A., Baggett H. (2008). *Bartonella tamiae* sp. nov., a newly recognized pathogen isolated from three human patients from Thailand. J. Clin. Microbiol..

[27] Heller R., Riegel P., Hansmann Y., Delacour G., Bermond D., Dehio C., Lamarque F., Monteil H., Chomel B., Piémont Y. (1998). *Bartonella tribocorum* sp. nov., a new *Bartonella* species isolated from the blood of wild rats. Int. J. Syst. Bacteriol..

[28] Welch D.F., Carroll K.C., Hofmeister E.K., Persing D.H., Robison D.A., Steigerwalt A.G., Brenner D.J. (1999). Isolation of a new subspecies, *Bartonella vinsonii* subsp. arupensis, from a cattle rancher: Identity with isolates found in conjunction with Borrelia burgdorferi and Babesia microti among naturally infected mice. J. Clin. Microbiol..

[29] Kosoy M., Murray M., Gilmore R.D.J., Bai Y., Gage K.L. (2003). *Bartonella* strains from ground squirrels are identical to *Bartonella washoensis* isolated from a human patient. J. Clin. Microbiol..

[30] Kosoy M., Bai Y., Sheff K., Morway C., Baggett H., Maloney S.A., Boonmar S., Bhengsri S., Dowell S.F., Sitdhirasdr A. (2010). Identification of *Bartonella* infections in febrile human patients from Thailand and their potential animal reservoirs. Am. J. Trop. Med. Hyg..

[31] Saengsawang P., Kaewmongkol G., Inpankaew T. (2021). Molecular Detection of *Bartonella* spp. and hematological evaluation in domestic cats and dogs from Bangkok, Thailand. Pathogens.

[32] Assarasakorn S., Veir J.K., Hawley J.R., Brewer M.M., Morris A.K., Hill A.E., Lappin M.R. (2012). Prevalence of *Bartonella* species, hemoplasmas, and *Rickettsia felis* DNA in blood and fleas of cats in Bangkok, Thailand. Res. Vet. Sci..

[33] Bai Y., Kosoy M.Y., Boonmar S., Sawatwong P., Sangmaneedet S., Peruski L.F. (2010). Enrichment culture and molecular identification of diverse *Bartonella* species in stray dogs. Vet. Microbiol..

[34] Saengsawang P., Kaewmongkol G., Phoosangwalthong P., Chimnoi W., Inpankaew T. (2021). Detection of zoonotic *Bartonella* species in ticks and fleas parasitizing free-ranging cats and dogs residing in temples of Bangkok, Thailand. Vet. Parasitol. Reg. Stud. Rep..

[35] Kosoy M., Bai Y. (2019). *Bartonella* bacteria in urban rats: A movement from the jungles of Southeast Asia to metropoles around the globe. Front. Ecol. Evol..

[36] Daniel W.W. (2013). Biostatistics: A Foundation for Analysis in the Health Sciences, 10e Student Solutions Manual.

[37] Norman A.F., Regnery R., Jameson P., Greene C., Krause D.C. (1995). Differentiation of *Bartonella*-like isolates at the species level by PCR-restriction fragment length polymorphism in the citrate synthase gene. J. Clin. Microbiol..

[38] Brown L.D., Cai T.T., DasGupta A. (2001). Interval estimation for a binomial proportion. Stat. Sci..

[39] R Core Team (2020). R: A Language and Environment for Statistical Computing.

[40] Herbreteau V., Bordes F., Jittapalapong S., Supputamongkol Y., Morand S. (2012). Rodent-borne diseases in Thailand: Targeting rodent carriers and risky habitats. Infect. Ecol. Epidemiol..

[41] Prompiram P., Poltep K., Pamonsupornvichit S., Wongwadhunyoo W., Chamsai T., Rodkvamtook W. (2020). Rickettsiae exposure related to habitats of the oriental house rat (*Rattus tanezumi*, Temminck, 1844) in Salaya suburb, Thailand. Int. J. Parasitol. Parasites Wildl..

[42] Morand S., Blasdell K., Bordes F., Buchy P., Carcy B., Chaisiri K., Chaval Y., Claude J., Cosson J.-F., Desquesnes M. (2019). Changing landscapes of Southeast Asia and rodent-borne diseases: Decreased diversity but increased transmission risks. Ecol. Appl..

[43] Billeter S.A., Sangmaneedet S., Kosakewich R.C., Kosoy M.Y. (2012). *Bartonella* species in dogs and their ectoparasites from Khon Kaen province, Thailand. S. Asian J. Trop. Med. Public Health.

[44] Malania L., Bai Y., Osikowicz L.M., Tsertsvadze N., Katsitadze G., Imnadze P., Kosoy M. (2016). Prevalence and diversity of *Bartonella* species in rodents from Georgia (Caucasus). Am. J. Trop. Med. Hyg..

[45] Kerkhoff F.T., Bergmans A.M., van Der Zee A., Rothova A. (1999). Demonstration of *Bartonella grahamii* DNA in ocular fluids of a patient with neuroretinitis. J. Clin. Microbiol..

[46] Blasdell K.R., Perera D., Firth C. (2019). High prevalence of rodent-borne *Bartonella* spp. in urbanizing environments in Sarawak, Malaysian Borneo. Am. J. Trop. Med. Hyg..

[47] Klangthong K., Promsthaporn S., Leepitakrat S., Schuster A.L., McCardle P.W., Kosoy M., Takhampunya R. (2015). The distribution and diversity of *Bartonella* species in rodents and their ectoparasites across Thailand. PLoS ONE.

[48] Kim K.S., Inoue K., Kabeya H., Sato S., Takada T., Pangjai D., Chiu S.-H., Fujita H., Kawabata H., Takada N. (2016). Prevalence and diversity of *Bartonella* species in wild small mammals in Asia. J. Wildl. Dis..

[49] Neves E.S., Mendenhall I.H., Borthwick S.A., Su Y.C.F., Smith G.J.D. (2018). Detection and genetic characterization of diverse *Bartonella* genotypes in the small mammals of Singapore. Zoonoses Public Health.

[50] Loan H.K., Van Cuong N., Takhampunya R., Klangthong K., Osikowicz L., Kiet B.T., Campbell J., Bryant J., Promstaporn S., Kosoy M. (2015). *Bartonella* species and trombiculid mites of rats from the Mekong Delta of Vietnam. Vector Borne Zoonotic Dis..

[51] Liu Q., Sun J., Lu L., Fu G., Ding G., Song X., Meng F., Wu H., Yang T., Ren Z. (2010). Detection of *Bartonella* species in small mammals from Zhejiang Province, China. J. Wildl. Dis..

[52] Winoto I.L., Goethert H., Ibrahim I.N., Yuniherlina I., Stoops C., Susanti I., Kania W., Maguire J.D., Bangs M.J., Telford S.R. (2005). *Bartonella* species in rodents and shrews in the greater Jakarta area. S. Asian J. Trop. Med. Public Health.

[53] Bai Y., Kosoy M.Y., Lerdthusnee K., Peruski L.F., Richardson J.H. (2009). Prevalence and genetic heterogeneity of *Bartonella* strains cultured from rodents from 17 provinces in Thailand. Am. J. Trop. Med. Hyg..

[54] Panthawong A., Grieco J.P., Ngoen-Klan R., Chao C.-C., Chareonviriyaphap T. (2020). Detection of *Anaplasma* spp. and *Bartonella* spp. from wild-caught rodents and their ectoparasites in Nakhon Ratchasima Province, Thailand. J. Vector Ecol..

[55] Böge I., Pfeffer M., Htwe N.M., Maw P.P., Sarathchandra S.R., Sluydts V., Piscitelli A.P., Jacob J., Obiegala A. (2021). First detection of *Bartonella* spp. in small mammals from rice storage and processing facilities in Myanmar and Sri Lanka. Microorganisms.

[56] Peterson A.C., Ghersi B.M., Alda F., Firth C., Frye M.J., Bai Y., Osikowicz L.M., Riegel C., Lipkin W.I., Kosoy M.Y. (2017). Rodent-borne *Bartonella* infection varies according to host species within and among cities. Ecohealth.

[57] Kosoy M., Mandel E., Green D., Marston E., Childs J. (2004). Prospective studies of *Bartonella* of rodents. Part I demographic and temporal patterns in population dynamics. Vector Borne Zoonotic Dis..

[58] Gutiérrez R., Vayssier-Taussat M., Buffet J.-P., Harrus S. (2017). Guidelines for the isolation, molecular detection, and characterization of *Bartonella* species. Vector Borne Zoonotic Dis..

[59] Himsworth C.G., Bai Y., Kosoy M.Y., Wood H., DiBernardo A., Lindsay R., Bidulka J., Tang P., Jardine C., Patrick D. (2015). An investigation of *Bartonella* spp., *Rickettsia typhi*, and Seoul hantavirus in rats (*Rattus* spp.) from an inner-city neighborhood of Vancouver, Canada: Is pathogen presence a reflection of global and local rat population structure?. Vector Borne Zoonotic Dis..

[60] Firth C., Bhat M., Firth M.A., Williams S.H., Frye M.J., Simmonds P., Conte J.M., Ng J., Garcia J., Bhuva N.P. (2014). Detection of zoonotic pathogens and characterization of novel viruses carried by commensal *Rattus norvegicus* in New York City. MBio.

[61] Gundi V.A.K.B., Davoust B., Khamis A., Boni M., Raoult D., La Scola B. (2004). Isolation of *Bartonella rattimassiliensis* sp. nov. and *Bartonella phoceensis* sp. nov. from European *Rattus norvegicus*. J. Clin. Microbiol..

[62] Gutiérrez R., Shalit T., Markus B., Yuan C., Nachum-Biala Y., Elad D., Harrus S. (2020). *Bartonella kosoyi* sp. nov. and *Bartonella krasnovii* sp. nov., two novel species closely related to the zoonotic *Bartonella elizabethae*, isolated from black rats and wild desert rodent-fleas. Int. J. Syst. Evol. Microbiol..

[63] Abreu-Yanes E., Abreu-Acosta N., Izquierdo-Rodriguez E., Martin-Carrillo N., Foronda P. (2020). *Bartonella* species and haplotypes in rodents and their fleas in Lanzarote and El Hierro in the Canary Islands, Spain. J. Vector Ecol..

[64] Theonest N.O., Carter R.W., Amani N., Doherty S.L., Hugho E., Keyyu J.D., Mable B.K., Shirima G.M., Tarimo R., Thomas K.M. (2019). Molecular detection and genetic characterization of *Bartonella* species from rodents and their associated ectoparasites from northern Tanzania. PLoS ONE.

[65] Kosoy M., McKee C., Albayrak L., Fofanov Y. (2018). Genotyping of *Bartonella* bacteria and their animal hosts: Current status and perspectives. Parasitology.

[66] Scola B.L., Zeaiter Z., Khamis A., Raoult D. (2003). Gene-sequence-based criteria for species definition in bacteriology: The *Bartonella* paradigm. Trends Microbiol..

[67] Paziewska A., Harris P.D., Zwolińska L., Bajer A., Siński E. (2011). Recombination within and between species of the alpha proteobacterium *Bartonella* infecting rodents. Microb. Ecol..

[68] Buffet J.-P., Pisanu B., Brisse S., Roussel S., Félix B., Halos L., Chapuis J.-L., Vayssier-Taussat M. (2013). Deciphering *Bartonella* diversity, recombination, and host specificity in a rodent community. PLoS ONE.

